# Bettseitige Bildgebung

**DOI:** 10.1007/s00063-024-01191-3

**Published:** 2024-10-09

**Authors:** Robert Zilberszac

**Affiliations:** Universitätsklinik für Innere Medizin II, Abteilung für Kardiologie, Medizinische Universität Wien, Währinger Gürtel 18–20, 1090 Wien, Österreich

**Keywords:** Echokardiographie, Volumenstatus, Hämodynamik, Linksventrikuläre Funktion, Rechtsventrikuläre Funktion, Echocardiography, Volume status, Hemodynamics, Ventricular function, left, Ventricular function, right

## Abstract

Die Sonographie, insbesondere die Echokardiographie, ist in der Beurteilung des Volumenstatus und der Hämodynamik kritisch kranker Patienten unerlässlich. Die Untersuchung des linken Ventrikels liefert neben einer Beurteilung der Ventrikelfunktion auch weitere wertvolle Informationen, einschließlich des „kissing papillary muscle sign“, das auf einen Volumenbedarf hinweisen kann. Die Untersuchung des rechten Ventrikels ist ebenfalls wichtig, da er sowohl auf Volumen- als auch auf Druckbelastung empfindlich reagiert. Die Beurteilung der diastolischen Funktion und die Messung der V.-cava-inferior-Weite und -Variabilität geben Hinweise auf die Vorlast des linken bzw. rechten Ventrikels. Die Messung des Schlagvolumens und des Herzzeitvolumens ermöglicht eine weitere Beurteilung der Hämodynamik und lässt auch eine Ermittlung der Schlagvolumenvariabilität zu.

## Abstract (en)

Sonography, in particular echocardiography, is essential in the assessment of volume status and hemodynamics in critically ill patients. Examination of the left ventricle, in addition to assessing ventricular function, provides valuable information, including the “kissing papillary muscle sign,” which may indicate fluid responsiveness. Examination of the right ventricle is also important because it is sensitive to both volume and pressure overload. Assessment of diastolic function and measurement of inferior vena cava width and variability provide clues to left and right ventricular preload, respectively. Measurement of stroke volume and cardiac output allows further assessment of hemodynamics and also permits determination of stroke volume variability.

Durch ihre mittlerweile flächendeckende Verfügbarkeit ist die Sonographie und hier insbesondere die Echokardiographie zu einem unerlässlichen Teil des Assessments von Hämodynamik und Volumenstatus kritisch kranker Patienten geworden. Limitationen dieser Verfahren sind unbestritten, allen voran die Untersucherabhängigkeit der Ergebnisse und die insbesondere im intensivmedizinischen Setting oft deutlich erschwerten Untersuchungsbedingungen, unter anderem mit Überdruckbeatmung, eingeschränkter Lagerungsfähigkeit und frischer Sternotomie. In geübten Händen können hier aber sehr wertvolle Erkenntnisse gewonnen werden, ohne wesentlichen Aufwand für sowohl Patient als auch Untersucher.

## Linker Ventrikel

**Abb. 1:**
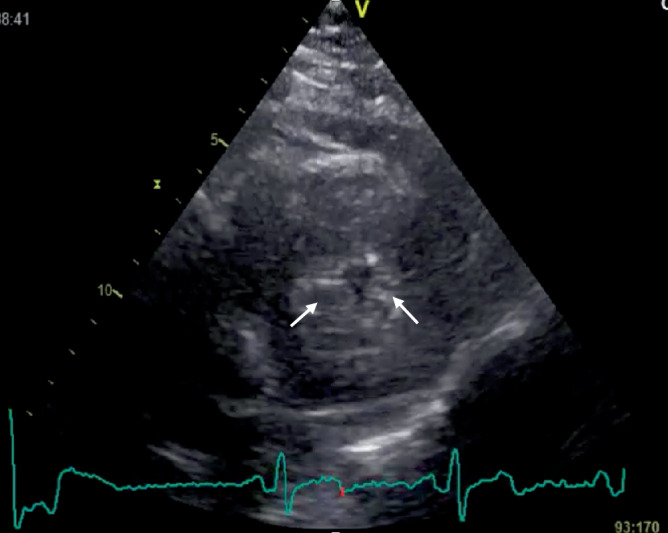
„Kissing papillary muscle sign“ bei einem Patienten mit hypertrophiertem linkem Ventrikel im Rahmen einer Aortenstenose. *Weiße Pfeile* Papillarmuskeln

Begonnen werden soll ein echokardiographisches Assessment des Volumenstatus idealerweise mit einer fundierten Untersuchung des linken Ventrikels. Schon durch die reine Betrachtung des Kontraktionsablaufs können wertvolle Erkenntnisse gewonnen werden – ein sogenanntes „kissing papillary muscle sign“, also die Berührung der Papillarmuskeln bzw. der freien Ventrikelwand und des Septums in der ventrikulären Systole spricht für ein kleines Cavum des linken Ventrikels und einen entsprechenden Volumenbedarf im Falle eines niedrigen Herzzeitvolumens (HZV) bzw. einer Hypotonie. Eine exakte Darstellung dieses Phänomens gelingt, neben der „oberflächlichen“ Beobachtung im apikalen 4‑Kammer-Blick, gut in der parasternalen kurzen Achse (Abb. 1) und kann hier durch eine zusätzliche Betrachtung im M‑Mode weiter objektiviert werden [1, 2].

Im intensivmedizinischen Alltag ist dieses Phänomen beispielsweise häufig bei Patienten nach Aortenklappenersatz wegen hochgradiger Aortenstenose zu beobachten. Oftmals besteht bei diesen Patienten durch die chronische Druckbelastung im Rahmen der Aortenstenose eine entsprechende konzentrische linksventrikuläre Hypertrophie, die in Kombination mit dem höheren Volumenbedarf im Setting des kardiochirurgischen Eingriffs zum oben genannten echokardiographischen Phänomen führt, gepaart mit dem entsprechenden Volumenbedarf bzw. einer „fluid responsiveness“.

Das „kissing papillary muscle sign“ lässt sich in der parasternalen kurzen Achse exakt darstellen

**Abb. 2:**
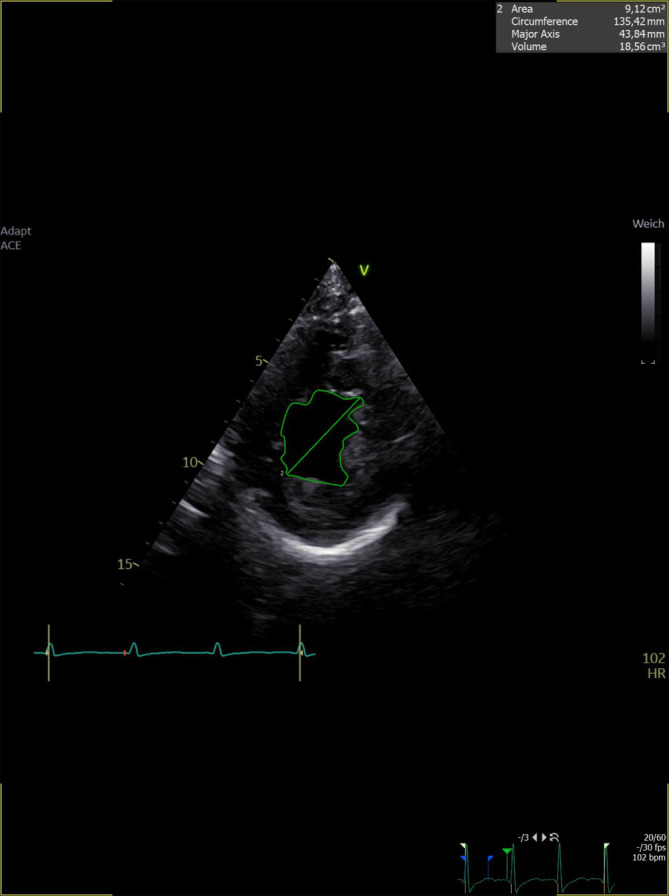
Messung der linksventrikulären enddiastolischen Fläche im parasternalen Kurzachsenschnitt

Der parasternale Kurzachsenschnitt bietet weiter auch die Möglichkeit, durch Umfahren des linksventrikulären Cavums am Ende der Diastole die linksventrikuläre enddiastolische Fläche zu messen (Abb. 2). Misst diese < 10 cm^2^, so ist dies bei Vorliegen konkordanter klinischer und apparativer Befunde als Zeichen eines möglichen Volumenbedarfs zu werten. Misst sie > 20 cm^2^, ist bei konkordanten klinischen und apparativen Befunden sowie nach Ausschluss einer schon vorbestehenden linksventrikulären Dilatation von einer möglichen Volumenüberladung auszugehen [1, 3].

**Abb. 3:**
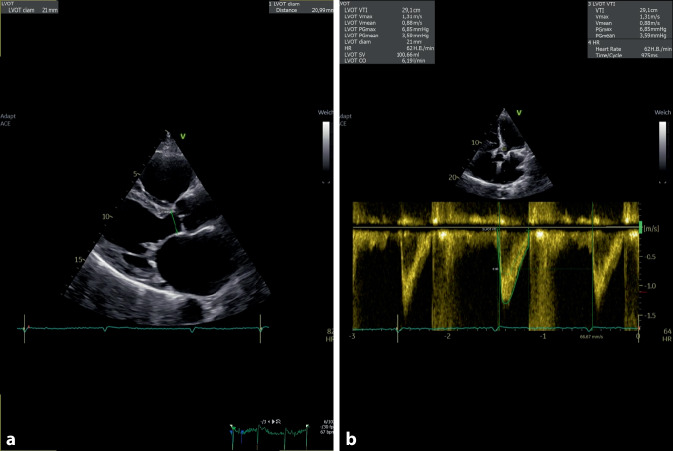
Bestimmung des Schlagvolumens bzw. Herzzeitvolumens (hier 6,2 l/min) durch Bestimmung des Durchmessers und des „velocity-time integral“ im linksventrikulären Ausflusstrakt. **a** Messung des LVOT Diameters in der parasternalen langen Achse, **b** Messung des VTI mittels PW Doppler

Eine Messung des Schlagvolumens (SV) bzw. des HZV kann echokardiographisch unter Zuhilfenahme der Volumenstromformel, die den Volumenstrom als Produkt des Querschnitts der Durchflussfläche und der Strömungsgeschwindigkeit berechnet, gewährleistet werden. Hierfür werden der Durchmesser des linksventrikulären Ausflusstrakts (LVOT), der hier anatomisch nicht ganz korrekt als Zylinder angenommen wird [4], und die Strömungsgeschwindigkeit (ausgedrückt als „velocity-time integral“ [VTI] im LVOT, gemessen mittels Pulsed-wave[PW]-Doppler) zu Hilfe genommen und in die Formel SV = Fläche LVOT · VTI LVOT eingesetzt. Multipliziert mit der Herzfrequenz kann nun das HZV bzw. bezogen auf die Körperoberfläche auch der Herzindex berechnet werden (Abb. 3).

Sobald das Schlagvolumen ermittelt wurde, kann echokardiographisch auch die Schlagvolumenvariabilität ermittelt werden. Dies ist einerseits über die oben genannte Formel möglich, andererseits aber auch vereinfacht durch die simple Messung der maximalen Flussgeschwindigkeit des größten und kleinsten Ausschlags des PW-Dopplerspektrums im LVOT über mehr als einen respiratorischen Zyklus. Als Cut-off für eine zu erwartende Flüssigkeitsresponsivität gilt auch hier der in der Pulskonturanalyse etablierte Cut-off von > 12 %. Auch die Limitationen gelten hier in Analogie zu jenen der Pulskonturanalyse: Die Patienten müssen kontrolliert beatmet (mit hohen Tidalvolumina) und im Sinusrhythmus sein; der intraabdominelle Druck sollte normal und der Thorax intakt sein [5].

**Abb. 4:**
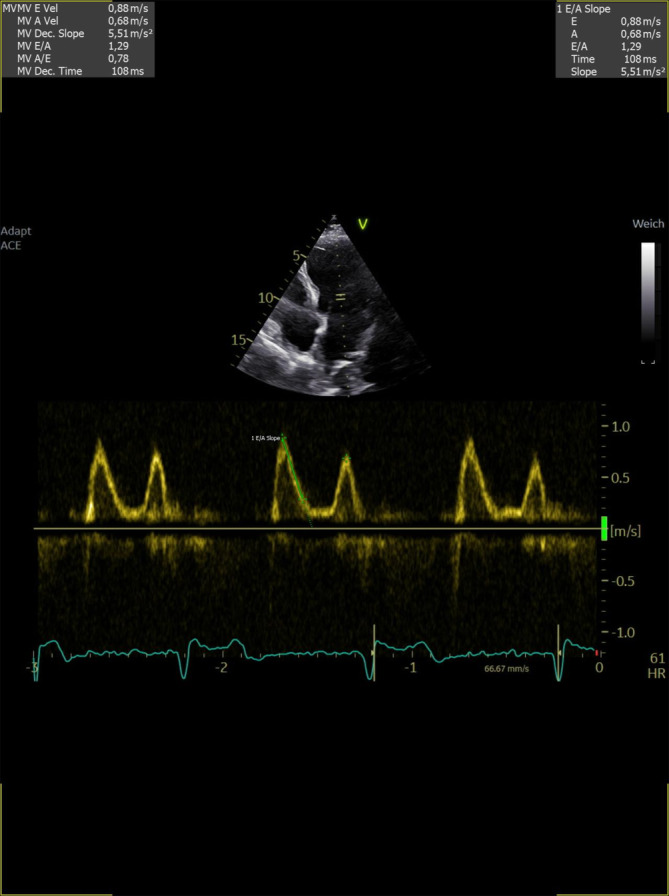
Bestimmung der diastolischen Funktion mittels Pulsed-wave-Doppler des Mitraleinstromsignals. Hier liegt ein pseudonormales Muster mit verkürzter Dezelerationszeit (108 ms) vor

Der Nachweis einer diastolischen Dysfunktion mit erhöhten Füllungsdrücken lässt Rückschlüsse auf die linksventrikuläre Vorlast – genauer: den linksventrikulären enddiastolischen Druck – zu, sofern keine intrinsische Reduktion der myokardialen Compliance vorliegt [6]. Ein (vereinfachtes) Assessment der diastolischen Funktion kann bei Patienten im Sinusrhythmus durch Messung des LV-Einstrom-Signals über die Mitralklappe mittels PW-Doppler gewährleitet werden. Beim Gesunden bestehen hier eine prädominant passive frühdiastolische Ventrikelfüllung (ausgedrückt als E‑Welle im PW-Dopplerspektrum) und eine aktive spätdiastolische Ventrikelfüllung (ausgedrückt als A‑Welle im PW-Dopplerspektrum). Bei einer etwas reduzierten Compliance des LV kommt es zur Relaxationsstörung und die A‑Welle wird größer als die E‑Welle. Dies ist aber auch bei völlig gesunden Menschen ein häufig anzutreffendes Muster und per se nicht mit erhöhten Füllungsdrücken assoziiert [7]. Kommt es allerdings zu einer Pseudonormalisierung, also einer Zunahme der E‑Welle aufgrund eines frühdiastolischen atrialen „Kicks“ bedingt durch einen erhöhten linksatrialen Druck (= „pulmonary capillary wedge pressure“ [PCWP]/linksventrikulärer enddiastolischer Druck [LVEDP]), so ist dies als Marker einer erhöhten Vorlast zu interpretieren (Abb. 4). Eine Abgrenzung von der normalen diastolischen Funktion ist hier durch das oftmalige bestehen einer linksatrialen Dilatation (der linke Vorhof ist das „Barometer“ des LV [8]) und durch den Nachweis einer Reduktion der frühdiastolischen Geschwindigkeit des Mitralanulus im Gewebsdoppler möglich [9]. Im Falle einer noch stärkeren Zunahme des E/A-Verhältnisses mit verkürzter Dezelerationszeit der E‑Welle (wiederum als Ausdruck des atrialen „Kicks“ bei hohem Druck im linken Vorhof) ist von einem restriktiven Füllungsmuster und entsprechend von einer noch höheren Vorlast auszugehen. Eine Pilotstudie mit 54 Intensivpatienten konnte zeigen, dass bei Patienten mit einem E/A-Verhältnis von > 2 und einer Dezelerationszeit der E‑Welle von < 160 ms der PCWP üblicherweise > 18 mm Hg liegt und dass der PCWP 20 mm Hg übersteigt, wenn die Dezelerationszeit < 120 ms beträgt [10]. Es sei allerdings nochmals erwähnt, dass dies nur gelten kann, wenn keine intrinsische myokardiale Compliancestörung vorliegt.

## Rechter Ventrikel

Kein echokardiographisches Assessment, insbesondere mit der Fragestellung des Volumenstatus, kann ohne eine Untersuchung des rechten Ventrikels (RV) auskommen. Hier ist aber insbesondere im intensivmedizinischen Kontext zu erwähnen, dass der RV nicht nur gegenüber einer akuten Volumenbelastung, sondern auch gegenüber einer akuten Druckbelastung sehr empfindlich ist; in beiden Fällen reagiert er mit einer Dilatation des Cavums und kann somit (abseits einer akuten Druckbelastung etwa bei einer Lungenembolie) auch im Rahmen erhöhter Beatmungsdrücke dilatieren. Da sich LV und RV ein fixiertes Kompartiment im Perikard miteinander teilen, muss in Gegenwart eines bereits vergrößerten RV bedacht werden, dass eine weitere Dilatation des RV im Rahmen zusätzlicher Flüssigkeitsgabe mit einer Beeinträchtigung des LV einhergehen wird. Ein definitives Zeichen dafür, dass eine Flüssigkeitssubstitution beendet werden sollte, ist jedenfalls ein dilatierter RV, dessen Schlagvolumen nach Flüssigkeitsgabe nicht mehr steigt [11].

## V. cava inferior

Zyklische Änderungen des intrathorakalen Drucks führen zu Änderungen des zentralen Venendrucks (ZVD), wodurch sich der venöse Rückfluss verändert. Im Falle einer kontrollierten Beatmung dehnt sich die V. cava inferior (VCI) bei Inspiration aus und kollabiert bei Exspiration. Diese Variation wird aufgehoben, wenn der ZVD hoch ist. Das Fehlen einer respiratorischen Variation ist als Anzeichen für ein fehlendes Ansprechen auf Volumengabe zu werten [12]. Im Gegensatz dazu, und ähnlich wie bei der LV-Schlagvolumenvariabilität, können große Variationen der VCI-Größe eine Volumenresponsivität voraussagen. Ein Grenzwert für die „Variabilität“ des Durchmessers von mehr als 12 % identifiziert hier potenzielle Responder [13]. Die Variation des VCI-Durchmessers wurde auch bei spontan atmenden Patienten untersucht, hier ist der prädiktive Wert jedoch schwächer [14].

Der Wert des zentralen Venendrucks als Marker des Volumenstatus ist stark umstritten

Was die statische Messung der VCI-Weite betrifft, so kann bei mechanisch beatmeten Patienten ein ZVD von < 10 mm Hg angenommen werden, wenn die VCI < 12 mm misst [15]. Bei spontan atmenden Patienten liegt der beste Cut-off-Wert für einen ZVD über oder unter 10 mm Hg bei 2 cm [16]. Bei invasiv beatmeten Patienten ist die VCI jedoch häufig aufgrund eines erhöhten intrathorakalen Drucks und nicht als Ausdruck des intravaskulären Volumenstatus erweitert, außerdem ist der Wert des ZVD als Marker des Volumenstatus stark umstritten [17].

## Fazit für die Praxis

- Die Echokardiographie ist in geübten Händen ein risikofreies und sehr aussagekräftiges Verfahren für das Assessment von Volumenstatus und Hämodynamik kritisch kranker Patienten.
- Naturgemäß können die echokardiographischen Verfahren insbesondere bei derartigen Fragestellungen nur hilfreich sein, wenn sie im Rahmen eines integrierten Vorgehens angewendet werden, das heißt unter Einbeziehung der Anamnese und klinischen Untersuchung.
